# Supplementary material for: Using secondary cases to characterize the severity of an emerging or re-emerging infection
Source: Nat Commun. 2021 Nov 4;12:6372. doi: 10.1038/s41467-021-26709-7 (PMC8569220; doi:10.1038/s41467-021-26709-7)
Supplement: Supplementary file 1 — Supplementary Information [file 41467_2021_26709_MOESM1_ESM.pdf]

Supplementary Information

for 'Using secondary cases to characterize the severity of an emerging or re-emerging infection'

Tim K. Tsang[1,2] PhD, Can Wang[1] MPH, Bingyi Yang[1] PhD, Simon Cauchemez[†3] PhD, Benjamin J. Cowling[†1,2] PhD

[†] These authors jointly supervised this work

Affiliations:

1. WHO Collaborating Centre for Infectious Disease Epidemiology and Control, School of Public Health, Li Ka Shing Faculty of Medicine, The University of Hong Kong, Hong Kong Special Administrative Region, China.
2. Laboratory of Data Discovery for Health Limited, Hong Kong Science and Technology Park, New Territories, Hong Kong.
3. Mathematical Modelling of Infectious Diseases Unit, Institut Pasteur, UMR2000, CNRS, Paris, France.

Corresponding author:

Benjamin J. Cowling, School of Public Health, Li Ka Shing Faculty of Medicine, The University of Hong Kong, 7 Sassoon Road, Pokfulam, Hong Kong

Tel: +852 3917 6711; Email: bcowling@hku.hk

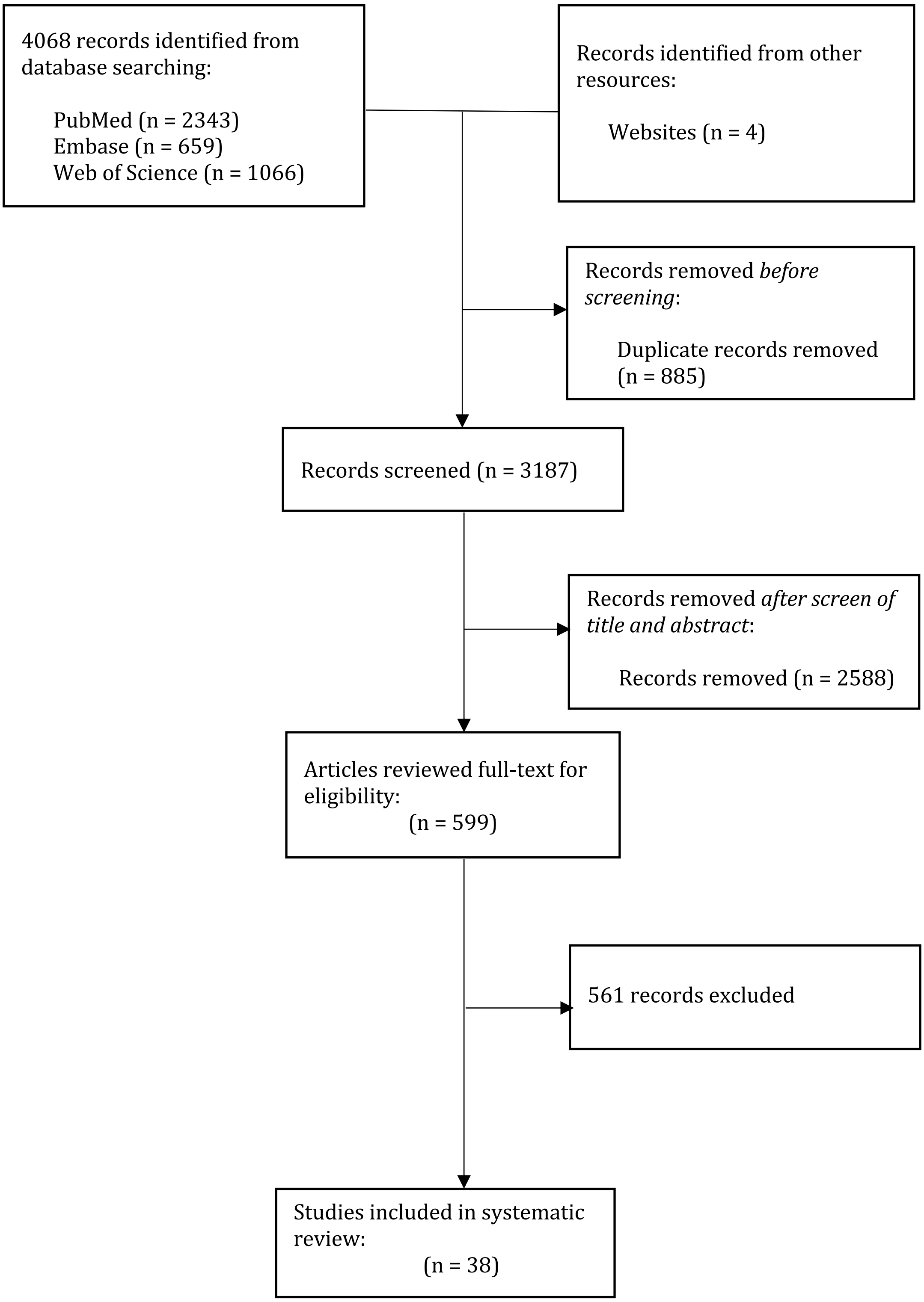

Supplementary Figure 1. Process of systematic review.

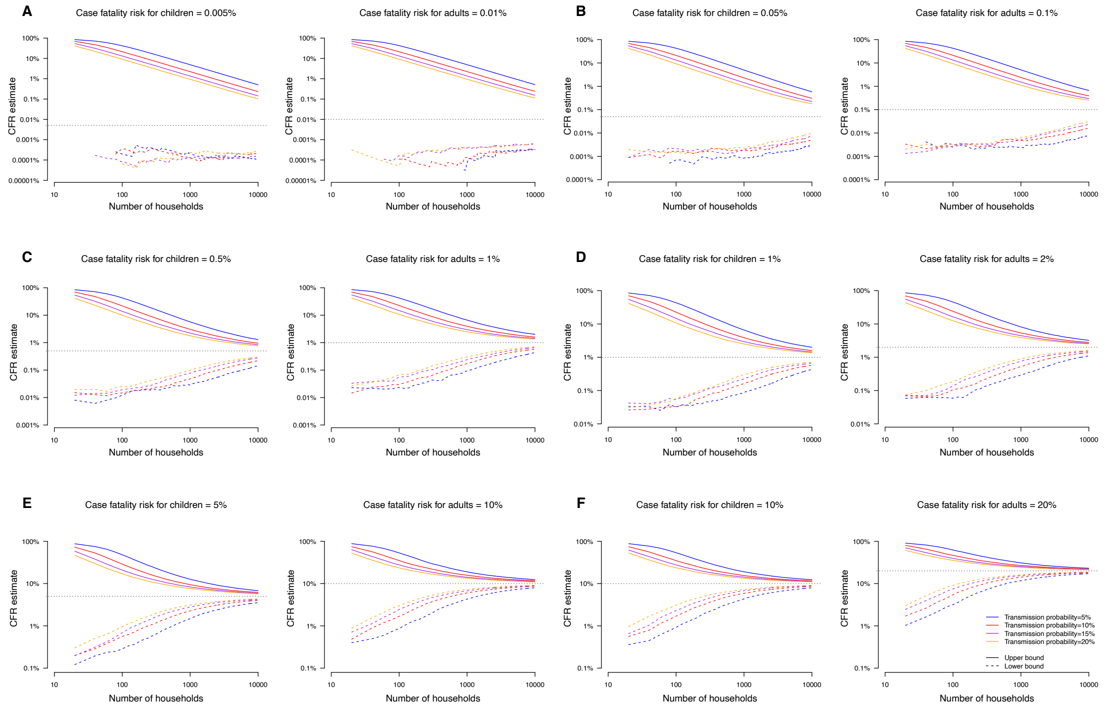

Supplementary Figure 2. The lower bound and upper bound of estimates for case fatality risk (CFR) under different value of CFR (Panel A-F), and different secondary infection risk in households.

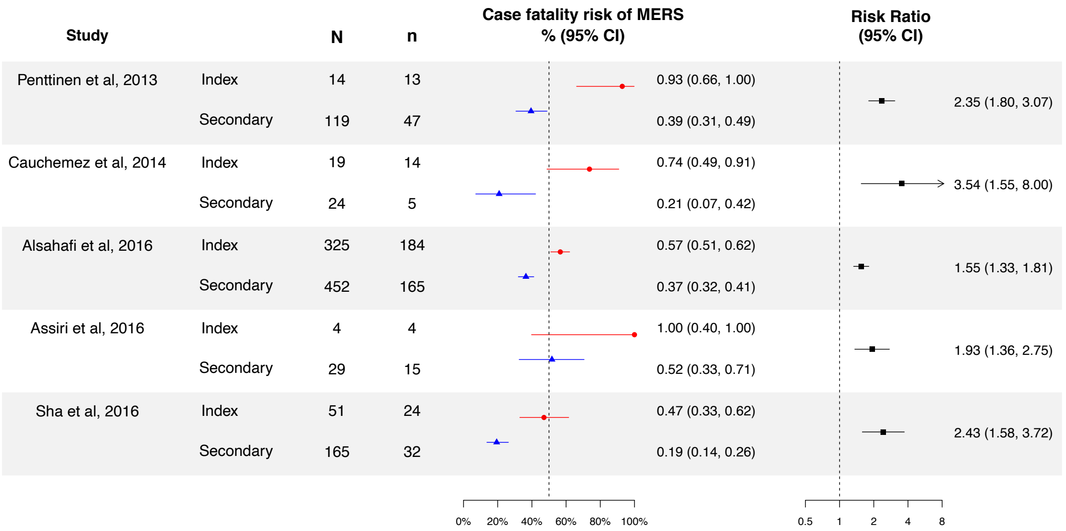

Supplementary Figure 3. Proportion of cases with death for the index cases and secondary cases for MERS, and their corresponding risk ratio of index cases, compared with secondary cases. Red circles and lines indicate the proportion of fever of index cases and the corresponding 95% exact binomial confidence intervals. Blue triangles and lines indicate the proportion of fever of secondary cases and the corresponding 95% confidence intervals. Black squares and lines indicate the risk ratio of proportion of fever of index cases compared with secondary cases and the corresponding 95% confidence intervals calculated based on normal approximation. All statistical tests are two-sided tests. Adjustments are not made for multiple comparisons.

Supplementary Table 1. Summary of included studies for pooled analysis.

Author (year)	*Location*	*Study period*	*Case ascertainment method*	*Test coverage of identified contacts*	*Contact settings*	*Severity measures*		*Remark*
						Types of symptoms	*Clinical severity*	
Arnedo-Pena *et al.* (2020)[1]	Castello, Spain	Feb 2020 to May 2020	Index: RT-PCR Secondary: RT-PCR & symptom-based	Only 39% of contacts were tested	Household	NA	Hospitalization, death	
Bi *et al.* (2020)[2]	Shenzhen, China	Jan 2020 to Feb2020	Index: RT-PCR Secondary: RT-PCR	All contacts were tested	Household, travel, meal	Fever, cough, chills, diarrhea, headache, sore throat and others	Case severity*, Symptom status	
Bo *et al.* (2021)[3]	Wuhan, China	Jan 2020 to Feb2020	Index: RT-PCR Secondary: RT-PCR	All contacts were tested	Household	Fever, cough, fatigue, myalgia, dyspnea, headache, sore throat and others	Symptom status, hospitalization	

Boddington *et al.* (2021)[4]	UK	Jan 2020 to Apr 2020	Index: symptom-based & radiology Secondary: symptom-based & radiology	NA	Household, health care settings	Fever, cough, sore throat, shortness of breath	NA	Case definition changed over study period
Broccia *et al.* (2021)[5]	Denmark	Feb 2020 to Oct 2020	Index: RT-PCR Secondary: RT-PCR	Only symptomatic contacts were tested before 19 May 2020 and All contacts were tested afterward	Household	NA	Death	Households with multiple index cases were removed. 64% of cases identified in period with testing on all contacts
Chaw *et al.* (2020)[6]	Brunei	Mar 2020 to Apr 2020	Index: RT-PCR Secondary: RT-PCR	All contacts were tested	Household, social, religious gathering,	Fever, cough, runny nose	Case severity*, Symptom status	

					workplace, school and others			
Chen P *et al.* (2020)[7]	Chongqing, China	Jan 2020 to Feb 2020	Index: RT-PCR Secondary: RT-PCR	NA	NA	Fever, cough, runny nose	Symptom status	
Chen Y *et al*. (2021)[8]	Zhejiang, China	Jan 2020 to Feb 2020	Index: RT-PCR or serology Secondary: RT-PCR & serology	NA	Household and school	NA	Symptom status	
Cheng *et al*. (2021)[9]	Taiwan	Jan 2020 to Mar 2020	Index: RT-PCR Secondary: RT-PCR	All household and hospital contacts were tested (symptomatic contacts were tested in other settings)	Household, health care settings and others	NA	Symptom status	

Dawson *et al.* (2020)[10]	Milwaukee, US	Mar 2020 to Apr 2020	Index: RT-PCR Secondary: RT-PCR	Only symptomatic contacts were tested	Household	Fever, cough, headache, chills, fatigue, myalgia, sore throat, diarrhea and others	NA	
Dupraz *et al*. (2021)[11]	Canton of Vaud, Switzerland	Feb 2020 to Jun 2020	Index: NAAT Secondary: Serology	All contacts were tested	Household and others	Fever, cough, headache, fatigue, myalgia, sore throat, diarrhea and others	Symptom status and hospitalization	
Freeman *et al.* (2020)[12]	Multiple countries	Apr 2020 to May 2020	Index: RT-PCR & serology Secondary: RT-PCR	NA	Hospital and others	Fever, cough, sore throat, headache, shortness of breath and malaise	Symptom status	COVID-19 cases developed pernio-like lesions acral skin were collected from multiple countries

Gomaa *et al.* (2021)[13]	Egypt	Apr 2020 to Oct 2020	Index: RT-PCR Secondary: RT-PCR & serology	All contacts were tested	Household	Fever, cough, shortness of breath, fatigue, sore throat, headache and others	Symptom status	
Hu P *et al.* (2021)[14]	Guangzhou, China	Jan 2020 to Mar 2020	Index: RT-PCR Secondary: RT-PCR	All contacts were tested	Household, social, transportation, health care settings and others	Fever, cough, sore throat and myalgia	Case severity*, Symptom status	Only clusters included children were included in the analysis
Hu S *et al.* (2021)[15]	Hunan, China	Jan 2020 to Apr 2020	Index: RT-PCR Secondary: RT-PCR	All contacts were tested	Household, social and others	NA	Case severity*, Symptom status	
Kuwelker *et al.* (2021)[16]	Bergen, Norway	Feb 2020 to Apr 2020	Index: RT-PCR Secondary: serology	All contacts were tested	Household	NA	Symptom status	

Li F *et al.* (2021)[17]	Wuhan, China	Dec 2019 to Apr 2020	Index: RT-PCR & clinical criteria† Secondary: RT-PCR & clinical criteria†	All contacts were tested	Household	Fever, cough, fatigue, myalgia, headache, chills, diarrhea, nausea and others	Case severity*, Symptom status	
Li W *et al.* (2020)[18]	Hubei, China	Jan 2020 to Feb 2020	Index: RT-PCR Secondary: RT-PCR	All contacts were tested	Household	Fever, cough, chills, fatigue, nausea, diarrhea and others	Symptom status	
Li J *et al.* (2020)[19]	Wuhan, China	Jan 2020 to Mar 2020	Index: RT-PCR & serology &clinical criteria† Secondary: RT-PCR & serology & clinical criteria†	All contacts were tested	Household	Fever, cough, fatigue, dyspnea, diarrhea and others	Case severity (without moderate and asymptomatic group)*	

Luo *et al.* (2020)[20]	Guangzhou, China	Jan 2020 to Mar 2020	Index: RT-PCR & Secondary: RT-PCR	All contacts were tested	Household, public transportation, workplace, health care settings and entertainment venues	Fever, cough, fatigue, myalgia, expectoration and diarrhea	Case severity (Only based on clusters with secondary cases)*, Symptom status	
Maltezou *et al.* (2021)[21]	Greece	Feb 2020 to May 2020	Index: RT-PCR Secondary: RT-PCR	94% of contacts were tested	Household	Fever, cough, headache, dyspnea, diarrhea, chills, vomit and others	Case severity*, Symptom status, death	
Martinez-Fierro *et al.* (2021)[22]	Zacatecas, Mexico	Jun 2020 to Jul 2020	Index: RT-PCR Secondary: RT-PCR	All contacts were tested	NA	Fever, cough, headache, dyspnea, diarrhea, chills, vomit and others	NA	
Miyahara *et al*. (2021)[23]	Japan	Feb 2020 to May 2020	Index: RT-PCR Secondary: RT-PCR	NA	Household	NA	Symptom status	

Reukers *et al.* (2021)[24]	Netherlands	Mar 2020 to May 2020	Index: RT-PCR Secondary: RT-PCR	All contacts were tested	Household	NA	Case severity*, Symptom status	
Salihefendic *et al*. (2021)[25]	Bosnia and Herzegovina	Mar 2020 to Dec 2020	Index: RT-PCR or serology or radiology Secondary: RT-PCR or serology or radiology	All contacts were tested	Household	NA	Hospitalization and death	
Sami *et al.* (2021)[26]	Illinois, US	Feb 2021	Index: NAAT or antigen Secondary: NAAT or antigen	52% of contacts were tested	Household, long-term care facilities, school	NA	Symptom status and hospitalization	
Shi *et al.* (2021)[27]	Wuxi, China	Jan 2020 to	Index: RT-PCR Secondary: RT-PCR	All contacts were tested	NA	NA	Case severity*, Symptom status	

		Mar 2020						
Soriano-Arandes *et al.* (2021)[28]	Catalonia, Spain	Jul 2020 to Oct 2020	Index: RT-PCR Secondary: RT-PCR	All contacts were tested	Household	NA	Symptom status and hospitalization	Only pediatric index and secondary cases were included in analysis
Steinberg *et al.* (2021)[29]	South Dakota, US	Mar 2020 to Apr 2020	Index: RT-PCR Secondary: RT-PCR	All contacts were tested	Household and workplace	NA	Symptom status, hospitalization, and death	
Sun *et al.* (2020)[30]	Zhejiang, China	Jan 2020 to Feb 2020	Index: RT-PCR Secondary: RT-PCR	All contacts were tested	Household	Fever, cough, sore throat, chills, fatigue myalgia and diarrhea	Symptom status	
Thiel *et al.* (2021)[31]	Liechtenstein	Mar 2020 to	Index: RT-PCR Secondary:	All contacts were tested	Household and workplace	NA	Symptom status,	

		Apr 2020	Serology				hospitalization and death	
Trunfio *et al*. (2021)[32]	Turin, Italy	Mar 2020 to Sep 2020	Index: RT-PCR Secondary: RT-PCR	57.8% of contacts were tested	Household	NA	Symptom status and hospitalization	
Ustunday *et al*. (2021)[33]	Turkey	Mar 2020 to Sep 2020	Index: RT-PCR Secondary: RT-PCR	All contacts were tested	Household	NA	Symptom status and hospitalization	
Wang *et al.* (2020)[34]	Wuhan, China	Jan 2020 to Feb 2020	Index: RT-PCR Secondary: RT-PCR	67% of contacts were tested	Household	Fever, cough, fatigue, myalgia, dyspnea and others	Symptom status	
Wu J *et al*. (2020)[35]	Zhuhai, China	Jan 2020 to Feb 2020	Index: RT-PCR Secondary: RT-PCR	All contacts were tested	Household	Fever, cough, sore throat, headache, shortness of breath, diarrhea and others	Symptom status	

Wu P *et al*. (2021)[36]	Guangdong, Zhejiang, Jiangsu, Hubei and Chongqing, China	Jan 2020 to Apr 2020	Index: RT-PCR Secondary: RT-PCR	All contacts were tested	Household, transportation and others	NA	Symptom status	
Xie *et al.* (2021)[37]	Beijing, China	Jan 2020 to Feb 2020	Index: RT-PCR Secondary: RT-PCR	All contacts were tested	Household	NA	Case severity* (without moderate group), Symptom status	
Zheng *et al.* (2020)[38]	Anhui, China	Jan 2020 to Apr 2020	Index: RT-PCR Secondary: RT-PCR	All contacts were tested	Unspecified	Cough, sore throat, dyspnea, headache, myalgia, fatigue and others	Case severity*, Symptom status	

Abbreviations: RT-PCR = reverse transcriptase polymerase chain reaction; ICU = intensive care unit; NAAT = nucleic acid amplification test

*Case severity included four categories: asymptomatic, mild, moderate and severe/critical.

†Clinical criteria include radiology, white-cell count or/and epidemiological link to confirmed cases.

Supplementary Table 2. Signs and symptoms reported by index cases and their contacts with laboratory-confirmed COVID-19 infection in studies. Risk ratios and their p-values are computed by fisher-exact test. 95% confidence intervals of risk ratio calculated based on normal approximation. All statistical tests are two-sided tests. Adjustments are not made for multiple comparisons.

Study	Case type	Fever	Cough	Sore throat	Headache	Diarrhea	Fatigue	Myalgia
Dawson 2020	Index	18/26 (69%)	22/26 (85%)	11/26 (42%)	18/26 (69%)	8/26 (31%)	10/26 (38%)	16/26 (62%)
	Secondary	9/16 (56%)	12/16 (75%)	7/16 (44%)	14/16 (88%)	3/16 (19%)	5/16 (31%)	8/16 (50%)
	Risk ratio	1.23 (0.74,2.03)	1.13 (0.81,1.56)	0.97 (0.47,1.98)	0.79 (0.58,1.09)	1.64 (0.51,5.30)	1.23 (0.51,2.95)	1.23 (0.69,2.19)
	p-value	0.511	0.454	1.000	0.270	0.485	0.746	0.531
Li W 2020	Index	56/105 (53%)	31/105 (30%)		1/105 (1%)	1/105 (1%)	20/105 (19%)	5/105 (5%)
	Secondary	36/64 (56%)	11/64 (17%)		1/64 (2%)	3/64 (5%)	4/64 (6%)	1/64 (2%)
	Risk ratio	0.95 (0.72,1.26)	1.72 (0.93,3.17)		0.61 (0.04,9.58)	0.20 (0.02,1.91)	3.05 (1.09,8.52)	3.05 (0.36,25.5)
	p-value	0.752	0.098		1.000	0.153	0.023	0.410
Luo 2020	Index	57/68 (84%)	42/68 (62%)			9/68 (13%)	17/68 (25%)	12/68 (18%)
	Secondary	55/121 (45%)	43/121 (36%)			1/121 (1%)	14/121 (12%)	5/121 (4%)
	Risk ratio	1.84 (1.48,2.30)	1.74 (1.28,2.36)			16.01 (2.07,123.73)	2.16 (1.14,4.11)	4.27 (1.57,11.61)
	p-value	<0.001	0.001			<0.001	0.024	0.003
Sun 2020	Index	115/149 (77%)	63/149 (42%)	24/149 (16%)		7/149 (5%)	14/149 (9%)	10/149 (7%)

	Secondary	110/240 (46%)	65/240 (27%)	27/240 (11%)		6/240 (2%)	20/240 (8%)	13/240 (5%)
	Risk ratio	1.68 (1.43,1.98)	1.56 (1.18,2.07)	1.43 (0.86,2.39)		1.88 (0.64,5.48)	1.13 (0.59,2.16)	1.24 (0.56,2.75)
	p-value	<0.001	0.003	0.216		0.258	0.716	0.660
Wang 2020	Index	55/85 (65%)	11/85 (13%)				8/85 (9%)	
	Secondary	30/47 (64%)	8/47 (17%)				4/47 (9%)	
	Risk ratio	1.01 (0.78,1.32)	0.76 (0.33,1.76)				1.11 (0.35,3.48)	
	p-value	1.000	0.607				1.000	
Wu 2020	Index	28/35 (80%)	23/35 (66%)	3/35 (9%)	7/35 (20%)	4/35 (11%)	11/35 (31%)	
	Secondary	28/48 (58%)	31/48 (65%)	10/48 (21%)	10/48 (21%)	7/48 (15%)	15/48 (31%)	
	Risk ratio	1.48 (1.04,2.16)	1.02 (0.74,1.40)	0.41 (0.12,1.39)	0.96 (0.41,2.27)	0.78 (0.25,2.47)	1.01 (0.53,1.92)	
	p-value	0.037	1.000	0.22	1.000	0.753	1.000	
Hu P 2021	Index	87/100 (87%)	46/100 (46%)	20/100 (20%)				16/100 (16%)
	Secondary	27/59 (46%)	16/59 (27%)	3/59 (5%)				2/59 (3%)
	Risk ratio	1.90 (1.43,2.54)	1.70 (1.06,2.71)	3.93 (1.22,12.67)				4.72 (1.12,19.81)

	p-value	<0.001	0.020	0.010				0.018
Martinez-Fierro 2021	Index	6/19 (32%)	5/19 (26%)	3/19 (16%)		4/19 (21%)	6/19 (32%)	5/19 (26%)
	Secondary	5/34 (15%)	10/34 (29%)	12/34 (35%)		6/34 (18%)	7/34 (21%)	13/34 (38%)
	Risk ratio	2.15 (0.75,6.11)	0.89 (0.36,2.23)	0.45 (0.14,1.39)		1.19 (0.38,3.71)	1.53 (0.60,3.91)	0.69 (0.29,1.64)
	p-value	0.173	1.000	0.205		1.000	0.507	0.547
Li F 2021	Index	2644/4061 (65%)	1545/4061 (38%)	218/4061 (5%)	386/4061 (10%)	328/4061 (8%)	1191/4061 (29%)	575/4061 (14%)
	Secondary	326/842 (39%)	231/842 (27%)	29/842 (3%)	50/842 (6%)	47/842 (6%)	134/842 (16%)	51/842 (6%)
	Risk ratio	1.68 (1.54,1.84)	1.39 (1.23,1.56)	1.56 (1.07,2.28)	1.60 (1.20,2.13)	1.45 (1.08,1.95)	1.84 (1.57,2.17)	2.34 (1.77,3.08)
	p-value	<0.001	<0.001	0.019	0.001	0.013	<0.001	<0.001
Chaw 2020	Index	9/19 (47%)	14/19 (74%)	9/19 (47%)				
	Secondary	33/52 (63%)	28/52 (54%)	33/52 (63%)				
	Risk ratio	0.75 (0.45,1.25)	1.37 (0.95,1.98)	0.75 (0.45,1.25)				
	p-value	0.279	0.176	0.279				
Bi 2020	Index	258/292 (88%)	127/292 (43%)	19/292 (7%)	51/292 (17%)	2/292 (1%)	45/292 (15%)	69/292 (24%)

	Secondary	62/87 (71%)	20/87 (23%)	4/87 (5%)	10/87 (11%)	1/87 (1%)	7/87 (8%)	6/87 (7%)
	Risk ratio	1.24 (1.08,1.43)	1.89 (1.26,2.84)	1.42 (0.49,4.05)	1.52 (0.81,2.86)	0.60 (0.05,6.49)	1.92 (0.90,4.09)	3.43 (1.54,7.62)
	p-value	<0.001	0.001	0.617	0.244	0.544	0.109	<0.001
Boddington 2021	Index	72/91 (79%)	75/91 (82%)	28/91 (31%)				
	Secondary	46/94 (49%)	72/94 (77%)	41/94 (44%)				
	Risk ratio	1.62 (1.28,2.04)	1.08 (0.93,1.25)	0.71 (0.48,1.04)				
	p-value	<0.001	0.366	0.094				
Chen 2020	Index	11/18 (61%)	9/18 (50%)		1/18 (6%)		4/18 (22%)	
	Secondary	21/72 (29%)	32/72 (44%)		6/72 (8%)		8/72 (11%)	
	Risk ratio	2.10 (1.25,3.51)	1.12 (0.66,1.91)		0.67 (0.09,5.19)		2.00 (0.68,5.91)	
	p-value	0.015	0.793		1.000		0.248	
Gomaa 2021	Index	22/23 (96%)	18/23 (78%)		18/23 (78%)	11/23 (48%)	20/23 (87%)	
	Secondary	32/88 (36%)	31/88 (35%)		30/88 (34%)	18/88 (20%)	31/88 (35%)	
	Risk ratio	2.63 (1.97,3.51)	2.22 (1.56,3.17)		2.30 (1.60,3.30)	2.34 (1.29,4.23)	2.47 (1.78,3.41)	
	p-value	<0.001	<0.001		<0.001	0.015	<0.001	
Li J 2020	Index	42/51 (82%)	28/51 (55%)			7/51 (14%)	23/51 (45%)	

	Secondary	41/59 (69%)	30/59 (51%)			6/59 (10%)	20/59 (34%)	
	Risk ratio	1.19 (0.96,1.46)	1.08 (0.76,1.54)			1.35 (0.48,3.76)	1.33 (0.83,2.12)	
	p-value	0.128	0.705			0.768	0.246	
Bo 2021	Index	142/171 (83%)	63/171 (37%)	10/171 (6%)	6/171 (4%)		59/171 (35%)	14/171 (8%)
	Secondary	34/45 (76%)	15/45 (33%)	2/45 (4%)	2/45 (4%)		12/45 (27%)	2/45 (4%)
	Risk ratio	1.10 (0.92,1.32)	1.11 (0.70,1.75)	1.32 (0.30,5.79)	0.79 (0.16,3.78)		1.29 (0.76,2.19)	1.84 (0.43,7.81)
	p-value	0.282	0.729	1.000	0.673		0.375	0.533
Freeman 2020	Index	9/23 (39%)	9/23 (39%)	5/23 (22%)	7/23 (30%)			
	Secondary	1/20 (5%)	3/20 (15%)	4/20 (20%)	4/20 (20%)			
	Risk ratio	7.83 (1.08,56.52)	2.61 (0.82,8.33)	1.09 (0.34,3.50)	1.52 (0.52,4.45)			
	p-value	0.011	0.099	1.000	0.501			
Zheng 2020	Index		86/132 (65%)	9/132 (7%)	9/132 (7%)		29/132 (22%)	13/132 (10%)
	Secondary		107/175 (61%)	6/175 (3%)	6/175 (3%)		18/175 (10%)	7/175 (4%)
	Risk ratio		1.07 (0.90,1.27)	1.99 (0.73,5.45)	1.99 (0.73,5.45)		2.14 (1.24,3.68)	1.67 (1.02,3.95)
	p-value		0.477	0.191	0.191		0.006	0.040
Dupraz 2021	Index	122/215 (57%)	157/215 (73%)	78/215 (36%)	136/215 (63%)	61/215 (28%)	184/215 (86%)	129/215 (60%)

	Secondary	76/172 (44%)	83/172 (48%)	55/172 (32%)	92/172 (53%)	39/172 (23%)	107/172 (62%)	74/172 (43%)
	Risk ratio	1.28 (1.05,1.58)	1.51 (1.27,1.80)	1.13 (0.86,1.50)	1.18 (1.00,1.41)	1.25 (0.88,1.77)	1.38 (1.21,1.56)	1.39 (1.14,1.71)
	p-value	0.018	<0.001	0.391	0.061	0.243	<0.001	0.001

Supplementary Table 3. Case severity reported by index cases and their contacts with SARS-CoV-2 infections in studies. Risk ratios and their p-values are computed by fisher-exact test. 95% confidence intervals of risk ratio calculated based on normal approximation. All statistical tests are two-sided tests. Adjustments are not made for multiple comparisons. The p-values in the last columns are computed by chi-squared test for all severity levels.

Study	Case type	Asymptomatic	Mild	Moderate	Severe/Critical	P-value
Luo 2020	Index	1/68 (1%)	5/68 (7%)	42/68 (62%)	20/68 (29%)	0.001
	Secondary	8/121 (7%)	18/121 (15%)	84/121 (69%)	11/121 (9%)	
	Risk ratio	0.22 (0.03,1.74)	0.49 (0.19,1.27)	0.89 (0.71,1.11)	3.24 (1.65,6.34)	
	p-value	0.160	0.166	0.335	<0.001	
Bi 2020	Index	8/292 (3%)	74/292 (25%)	180/292 (62%)	30/292 (10%)	<0.001
	Secondary	17/87 (20%)	1/87 (1%)	66/87 (76%)	3/87 (3%)	
	Risk ratio	0.14 (0.06,0.31)	22.05 (3.11,156.31)	0.81 (0.70,0.94)	2.98 (0.93,9.53)	
	p-value	<0.001	<0.001	0.015	0.051	
Hu P 2021	Index	0/100 (0%)	27/100 (27%)	66/100 (66%)	7/100 (7%)	0.087
	Secondary	1/59 (2%)	25/59 (42%)	30/59 (51%)	3/59 (5%)	
	Risk ratio	0.20* (0.01,4.77)	0.64 (0.40,1.00)	1.30 (0.97,1.73)	1.38 (0.37,5.12)	

	p-value	NC	0.055	0.067	0.746	
Li F 2021	Index	567/29578 (2%)	14928/29578 (50%)	8416/29578 (28%)	5667/29578 (19%)	<0.001
	Secondary	439/10367 (4%)	5398/10367 (52%)	3088/10367 (30%)	1442/10367 (14%)	
	Risk ratio	0.45 (0.40,0.51)	0.97 (0.95,0.99)	0.96 (0.92,0.99)	1.38 (1.31,1.45)	
	p-value	<0.001	0.005	0.01	<0.001	
Hu S 2021	Index	159/1178 (13%)	299/1178 (25%)	570/1178 (48%)	150/1178 (13%)	<0.001
	Secondary	104/471 (22%)	153/471 (32%)	174/471 (37%)	40/471 (8%)	
	Risk ratio	0.61 (0.49,0.76)	0.78 (0.66,0.92)	1.31 (1.15,1.49)	1.50 (1.08,2.09)	
	P-value	<0.001	0.004	<0.001	0.017	
Reukers 2021	Index	0/55 (0%)	21/55 (38%)	11/55 (20%)	22/55 (40%)	<0.001
	Secondary	6/75 (8%)	52/75 (69%)	8/75 (11%)	9/75 (12%)	
	Risk ratio	0.11* (0.01,1.89)	0.55 (0.38,0.80)	1.88 (0.81,4.35)	3.33 (1.67,6.67)	
	P-value	NC	0.001	0.208	<0.001	
Chaw 2020	Index	4/19 (21%)	12/19 (63%)	2/19 (11%)	1/19 (5%)	0.476

	Secondary	5/52 (10%)	40/52 (77%)	5/52 (10%)	2/52 (4%)	
	Risk ratio	2.19 (0.66,7.31)	0.82 (0.56,1.19)	1.09 (0.23,5.18)	1.37 (0.13,14.24)	
	P-value	0.236	0.364	1.000	1.000	
Maltezou 2021	Index	0/23 (0%)	15/23 (65%)	6/23 (26%)	2/23 (9%)	0.002
	Secondary	16/45 (36%)	21/45 (47%)	7/45 (16%)	1/45 (2%)	
	Risk ratio	0.06* (0.01,3.15)	1.40 (0.91,2.15)	1.68 (0.64,4.41)	3.91 (0.37,40.92)	
	P-value	0.001	0.201	0.339	0.263	
Shi 2020	Index	4/46 (9%)	10/46 (22%)	26/46 (57%)	6/46 (13%)	0.131
	Secondary	6/23 (26%)	7/23 (30%)	9/23 (39%)	1/23 (4%)	
	Risk ratio	0.33 (0.10,1.07)	0.71 (0.31,1.63)	1.44 (0.82,2.55)	3.00 (0.38,23.47)	
	P-value	0.073	0.555	0.208	0.411	
Zheng 2020	Index	0/132 (0%)	9/132 (7%)	114/132 (86%)	9/132 (7%)	0.036
	Secondary	0/175 (0%)	11/175 (6%)	135/175 (77%)	29/175 (17%)	
	Risk ratio	1.32* (0.00,5.45)	1.08 (0.46,2.54)	1.12 (1.01,1.24)	0.41 (0.20,0.84)	

	P-value	1.000	1.000	0.055	0.013	

*added 0.5 for each cell

Supplementary Table 4. Summary of symptoms and clinical severity for COVID-19, restricted on studies with testing on all contacts of index cases. Overall, 13, 14, 22, 6 and 4 studies were included for fever, cough, symptom status, hospitalization and death respectively. 95% confidence intervals for proportion are computed by exact binomial method. Risk ratios, their 95% confidence intervals and p-values are computed by random effects meta-analyses with using the inverse variance method and restricted maximum likelihood estimator for heterogeneity. All statistical tests are two-sided tests. Adjustments are not made for multiple comparisons.

	Index cases	Secondary cases	Risk ratio (95% CI)	p-value	Heterogeneity (I^2)
Symptom					
Fever	3588/5308; 67.6% (66.3%, 68.9%)	865/1911; 45.3% (43%, 47.5%)	1.43 (1.21, 1.69)	<0.001	88.02
Cough	2248/5440; 41.3% (40%, 42.6%)	721/2086; 34.6% (32.5%, 36.6%)	1.41 (1.24, 1.6)	<0.001	61.48
Sore throat	393/5193; 7.6% (6.9%, 8.3%)	181/1754; 10.3% (8.9%, 11.8%)	1.2 (0.91, 1.57)	0.203	40.07
Headache	614/5034; 12.2% (11.3%, 13.1%)	201/1521; 13.2% (11.6%, 15%)	1.49 (1.15, 1.93)	0.003	50.92
Diarrhea	434/5018; 8.6% (7.9%, 9.5%)	134/1755; 7.6% (6.4%, 9%)	1.43 (1.18, 1.74)	<0.001	0
Fatigue	1619/5321; 30.4% (29.2%, 31.7%)	389/1975; 19.7% (18%, 21.5%)	1.66 (1.39, 1.97)	<0.001	52.63
Myalgia	869/5335; 16.3% (15.3%, 17.3%)	213/1927; 11.1% (9.7%, 12.5%)	1.96 (1.48, 2.59)	<0.001	60.15

Clinical Severity					
Case severity					
Asymptomatic	743/31468; 2.4% (2.2%, 2.5%)	586/11430; 5.1% (4.7%, 5.5%)	0.42 (0.22, 0.78)	0.006	88.85
Mild	15385/31468; 48.9% (48.3%, 49.4%)	5705/11430; 49.9% (49%, 50.8%)	0.8 (0.66, 0.96)	0.016	64.39
Moderate	9427/31468; 30% (29.5%, 30.5%)	3599/11430; 31.5% (30.6%, 32.3%)	1.07 (0.93, 1.25)	0.348	85.63
Severe/Critical	5912/31468; 18.8% (18.4%, 19.2%)	1540/11430; 13.5% (12.9%, 14.1%)	1.67 (1.02, 2.75)	0.043	85.01
Proportion of symptomatic cases	33011/34013; 97.1% (96.9%, 97.2%)	12338/13508; 91.3% (90.9%, 91.8%)	1.1 (1.03, 1.18)	0.004	98.71
Proportion of hospitalization	104/1377; 7.6% (6.2%, 9.1%)	46/1395; 3.3% (2.4%, 4.4%)	2.74 (1.41, 5.35)	0.003	60.36
Proportion of death	17/1100; 1.5% (0.9%, 2.5%)	4/388; 1% (0.3%, 2.6%)	3.77 (1.36, 10.48)	0.011	0

Supplementary Table 5. Summary of symptoms and clinical severity for COVID-19, restricted on studies with testing on all contacts of index cases, and using PCR to confirm both index and secondary cases. Overall, 9, 10 and 15 studies were included for fever, cough and symptom status respectively. 95% confidence intervals for proportion are computed by exact binomial method. Risk ratios, their 95% confidence intervals and p-values are computed by random effects meta-analyses with using the inverse variance method and restricted maximum likelihood estimator for heterogeneity. All statistical tests are two-sided tests. Adjustments are not made for multiple comparisons.

	Index cases	Secondary cases	Risk ratio (95% CI)	p-value	Heterogeneity (I^2)
Symptom					
Fever	758/958; 79.1% (76.4%, 81.7%)	390/750; 52% (48.4%, 55.6%)	1.35 (1.11, 1.65)	0.003	82.63
Cough	500/1090; 45.9% (42.9%, 48.9%)	346/925; 37.4% (34.3%, 40.6%)	1.37 (1.16, 1.61)	<0.001	51.28
Sore throat	97/917; 10.6% (8.7%, 12.8%)	97/740; 13.1% (10.8%, 15.8%)	1.13 (0.71, 1.79)	0.603	51.69
Headache	74/735; 10.1% (8%, 12.5%)	29/419; 6.9% (4.7%, 9.8%)	1.32 (0.86, 2.04)	0.204	0
Diarrhea	27/668; 4% (2.7%, 5.8%)	24/594; 4% (2.6%, 6%)	1.28 (0.55, 2.98)	0.566	46.66
Fatigue	201/971; 20.7% (18.2%, 23.4%)	97/814; 11.9% (9.8%, 14.3%)	1.59 (1.24, 2.04)	<0.001	8.48

Myalgia	144/1036; 13.9% (11.8%, 16.2%)	49/825; 5.9% (4.4%, 7.8%)	2.14 (1.28, 3.59)	0.004	48.19
Clinical Severity					
Case severity					
Asymptomatic	176/1890; 9.3% (8%, 10.7%)	147/1063; 13.8% (11.8%, 16.1%)	0.4 (0.18, 0.9)	0.026	70.06
Mild	457/1890; 24.2% (22.3%, 26.2%)	307/1063; 28.9% (26.2%, 31.7%)	0.75 (0.65, 0.86)	<0.001	3.46
Moderate	1011/1890; 53.5% (51.2%, 55.8%)	511/1063; 48.1% (45%, 51.1%)	1.11 (0.93, 1.32)	0.26	77.95
Severe/Critical	245/1890; 13% (11.5%, 14.6%)	98/1063; 9.2% (7.5%, 11.1%)	1.75 (0.96, 3.18)	0.066	74.02
Proportion of symptomatic cases	3569/3992; 89.4% (88.4%, 90.3%)	2083/2757; 75.6% (73.9%, 77.1%)	1.08 (1.01, 1.16)	0.02	96.53

Supplementary Table 6. Signs and symptoms reported by index cases and their household contacts with PCR-confirmed pandemic influenza A(H1N1) virus infection. Risk ratios and their p-values are computed by fisher-exact test. 95% confidence intervals of risk ratio calculated based on normal approximation. All statistical tests are two-sided tests. Adjustments are not made for multiple comparisons.

	Ip 2015				Papenburg 2010				Suess 2010			
	Index cases	Secondary cases	Risk Ratio	p-value	Index cases	Secondary cases	Risk Ratio	p-value	Index cases	Secondary cases	Risk Ratio	p-value
Number of participants	103	33			43	45			36	15		
Cough	97 (94%)	21 (64%)	1.48 (1.14,1.92)	<0.001	43 (100%)	38 (84%)	1.18 (1.04,1.34)	0.012	34 (94%)	10 (67%)	1.42 (1.04,2.29)	0.018
Fever	71 (69%)	16 (48%)	1.42 (1.02,2.21)	0.039	37 (86%)	30 (67%)	1.29 (1.02,1.64)	0.045	29 (81%)	8 (53%)	1.51 (0.92,2.49)	0.083
Sore throat	75 (73%)	15 (45%)	1.60 (1.08,2.37)	0.006					20 (56%)	3 (20%)	2.78 (1.25,Inf)*	0.030
Headache	50 (49%)	13 (39%)	1.23 (0.77,1.97)	0.425					27 (75%)	4 (27%)	2.81 (1.19,6.65)	0.002
Myalgia	39 (38%)	12 (36%)	1.04 (0.62,1.74)	1.000					27 (75%)	4 (27%)	2.81 (1.19,6.65)	0.002

Diarrhoea					36 (84%)	11 (24%)	3.42 (2.02,5.82)	<0.001				
Nausea					8 (19%)	7 (16%)	1.20 (0.47,3.01)	0.781				
Runny nose	90 (87%)	20 (61%)	1.44 (1.08,1.92)	0.002								
Sputum	91 (88%)	16 (48%)	1.82 (1.27,2.61)	<0.001								

*Due to small sample size, the upper bound cannot be estimated

Supplementary Table 7. Comparison of the estimates of severity based on secondary cases, and the literature.

	Severity estimates from our study	Chang et al. [39]	Chen et al. [40]	Ciapponi et al. [41]	Fathi et al. [42]	Islam et al. [43]	Li et al. [44]	Panahi et al. [45]	Wong et al. [46]	Cui et al. [47]	Hashan et al. [48]	Wang et al. [49]
Remark		Restricted to children	Based on Serology studies	Restricted to pregnant women			Restricted to children	Restricted to children		Restricted to children	Restricted to aged care facilities	Restricted to medical staff
Symptom												
Fever	45%	59%		28-100%	79%	59%	48%	96%	72%	51%	49%	
Cough	36.3%	46%			60%	47%	42%	91%	56%	41%	45%	
Sore throat	12.4%					9%			16%		5%	
Headache	13.8%								11%		4%	
Diarrhea	7.7%				11%						12%	
Fatigue	19.2%				33%						14%	
Myalgia	11.1%				33%				22%		3%	
Clinical Severity												
Case severity												

Asymptomatic	5.2%											
Mild	49.9%											
Moderate	31.4%											
Severe/Critical	13.4%	2%						6%				
Proportion of symptomatic cases	90.8%	74%	2-13%				77%			80%	70%	
Proportion of hospitalization	6.9%										37%	
Proportion of death	0.5%											6%

Supplementary Table 8. Probability densities for the distribution of the incubation period and Relative infectivity during the infectious period

	Days from infection to symptom onset													
Mean Duration (Days)	1	2	3	4	5	6	7	8	9	10	11	12	13	14
5	0.058	0.11	0.14	0.16	0.15	0.13	0.1	0.068	0.044	0.026	0.14	0.0072	0.0034	0.0015

	Days from symptom onset tor peak infectivity day														
Max duration (Days)	-5~2	3	4	5	6	7	8	9	10	11	12	13	14	15	16
22	1	1	1	1	1	0.8	0.8	0.6	0.6	0.4	0.4	0.3	0.3	0.1	0.1

Supplementary Note 1: Definition of case severity of COVID-19 in China

Severity of the disease of COVID-19 includes 4 categories: mild, moderate, severe and critical.

Mild cases were those who had mild symptoms and no sign of pneumonia on chest imaging.

Moderate cases are those who had fever and respiratory symptoms and signs of pneumonia.

Severe cases were those who meet any of the falling criteria: 1) Shortness of breath, RR ≥30times/min; 2) Oxygen saturation ≤93% at rest; 3) Alveolaroxygen partial pressure/fraction of inspiration O2 (PaO2/FiO2) ≤300 mmHg(1mmHg=0.133kPa).

Critical cases are those who meet any of the falling conditions: 1) respiratory failure requiring mechanical ventilation; 2) Shock; 3) Patients combined with other organ failure needed ICU monitoring and treatment.

While in the original document there were only 4 categories, in most studies, cases with no symptom would be classified as asymptomatic cases. Therefore, there could be 5 categories.

In our analysis, we combined severe and critical cases to a group to increase the statistical power.

Supplementary Note 2: Severity difference among index cases and secondary cases for other diseases

Higher severity for index cases than secondary cases was also observed for other infectious disease. For influenza A(H1N1)pdm09 [50-52], the frequency of symptoms in secondary cases was generally significantly lower than in index cases (Figure 1, Table S3). All studies reported higher frequency of cough for index cases than secondary cases, while two studies reported higher frequency of fever and sore throat for index cases than secondary cases. Five studies that reported the risk of death among index cases and secondary cases for MERS [53-57]. All studies identifying cases from symptomatic persons only, and hence this proportion should be considered as CFR. Four studies [53, 55-57] reported significantly higher CFR for index cases (range: 47% to 100%) than for secondary cases (19% to 52%) (Figure 3).

Supplementary Note 3: Estimating number of undetected index cases based on differences in severity profile between index cases and secondary cases.

Overview

For different infectious disease, the severity profile for index cases were more severe than secondary cases, likely due to case ascertainment bias, since severe cases could be detected with higher probability, compared with less severe cases. Here, we aim to develop a statistical model to infer the number of undetected index cases in households, by assuming the severe profile of secondary cases is the true severity profile for a disease.

Multinomial Model

Assume in a region and in a period, there were N observed index cases in households. The information of severity level was available for n of N observed index cases.

Assume there were *J* severity groups (in decreasing order and hence severity group 1 is the most severe one). Denote n_{j1} and n_{j2} the observed number of index cases and secondary cases respectively. Hence $n_1 = \sum_j n_{j1}$, and $n_2 = \sum_j n_{j2}$.

Denote P_j the proportion of index cases with severity level *j*, then:

$$n_{j1} \sim multinomial(n_1, P_1, P_2, \dots, P_J)$$

Denote p_j the proportion of secondary cases with severity level *j*, then:

$$n_{j2} \sim multinomial(n_2, p_1, p_2, \dots, p_J)$$

Hence, after estimating the posterior distribution of $P_1, P_2, \dots, P_J$ and $p_1, p_2, \dots, p_J$, we estimated the total number of index cases as follows:

First, we randomly draw a parameter vector $\widehat{P_1}, \widehat{P_2}, \dots, \widehat{P_J}$ from the posterior distribution of $P_1, P_2, \dots, P_J$, to simulate the distribution of severity levels of the observed index cases without information of severity levels (*N*-*n*), so that we may obtain the number of observed index cases in each severity group, $\widehat{n_{j1}}'$, where $N = \sum_j \widehat{n_{j1}}'$.

Denote the number of index cases for severity group j is $\widehat{N_{J1}}$. Assuming all index cases in the most severity group are detected, $\widehat{N_{11}} = \widehat{n_{11}'}$. For other groups, we first randomly draw a parameter vector $\widehat{p_1}, \widehat{p_2}, \dots, \widehat{p_J}$ from the posterior distribution of $p_1, p_2, \dots, p_J$, then we have

$$\widehat{N_{J1}} = \frac{\widehat{N_{11}}}{\widehat{p_1}} * \widehat{p_J}$$

for $j = 2,3,4$.

The proportion of missed index cases for each group j, $p_{miss,j}$, can be computed as

$$p_{miss,j} = 1 - \frac{\widehat{n_{J1}'}}{\widehat{N_{J1}}}$$

We repeated this 10000 times, and reported the mean, 2.5% and 97.5% quantile across these replications to obtain the estimate and the 95% credible intervals of each parameter of interest, $\widehat{n_{J1}'}$, $\widehat{N_{J1}}$ and $p_{miss,j}$ for each j.

Likelihood function

For $P_1, P_2, \dots, P_J$ and $p_1, p_2, \dots, p_J$ they followed a standard multinomial distribution.

Priors

We will use Dirichlet prior for the parameter with $\alpha_1, \alpha_2, \dots, \alpha_J$, where $\alpha_j = 0.5$ for all j, for the multinomial distribution, to represent uninformative priors (Jeffery's prior).

Inference

For each MCMC step, we first update the parameter $P_1, P_2, \dots, P_J$ and $p_1, p_2, \dots, p_J$, by using the Gibbs sampler. For P_j, the full conditional distribution is:

$$Dirichlet(n_{j1} + \alpha_j)$$

For p_j, the full conditional distribution is:

$$Dirichlet(n_{j2} + \alpha_j)$$

After that, we can estimate $\widehat{n_{j1}}'$, $\widehat{N_{j1}}$ and $p_{miss,j}$, according to Section 1.2.

Supplementary Note 4: Simulation study to determine the sample size requirement to use secondary cases to estimate severity profile.

Overview

There could be detection bias in index cases since severe cases are more likely to be detected compared with mild/asymptomatic cases. However, after detection of index cases, their contacts are actively followed in contact tracing studies, such as testing regardless of presence of symptoms. Therefore, the severity profile of secondary cases could be unbiased estimate of the disease severity profile. We aim to conduct simulation studies to determine the sample size requirement for using severity profile of secondary cases to estimate disease severity profile. In the simulation, we use household study as an example of contact tracing study to conduct sample size calculation for severity profile. We use case fatality risk (CFR) as an example to illustrate this.

Household transmission model

Let $p_j(t)$ be the probability of infection at day *t* for individual *j*, then:

$p_j(t) = 1 - (1 - b) * \prod_i (1 - p'_{ij}(t))$,

where *b* is the infection probability from community, and $p'_{ij}(t)$ is the effective probability of transmission from individual *i* to *j* at day *t*:

$$logit\left(p'_{ij}(t)\right) = logit\left(p_{ij}\right) + \boldsymbol{\beta X_{ij}(t)}$$

where *logit(y)=log(y/(1-y)),* and $X_{ij}(t)$ indicate the factors affecting susceptibility or infectivity. In our simulation, we do not include factors affecting susceptibility or infectivity. Therefore, $p'_{ij}(t) = p$ for all *i* and *j*.

Based on this, we can simulate the infection status for each household member for each day. When they are infected, we also simulate the fatality outcome with probability p_d.

Natural history and household transmission probability of COVID-19

We assumed the infectivity was peak at the symptom onset for symptomatic cases. For asymptomatic cases, we assume there was a pseudo symptom onset time which was the peak infectivity.

As summarized in Jing et al. [58] and other reports [59, 60], we fit the model with an incubation period with mean 5 days, and relative infectiousness profile with 21 days. We summarized this information in Table S1.

We used the same approach in Jing et al. to compute the transmission probability for close contact by contact type. Hence transmission probability can be computed as

$$Transmission\,probability = 1 - \prod_{l=D_{min}}^{D_{max}} \left(1 - p_k^*(l)\phi(l)\right)$$

where $p_k^*(l)$ is the transmission probability by contact type estimated from model without covariates, and $\phi(l)$ is the infectious profile. Based on this, we can control the transmission probability in the simulation.

Simulation details

We will use an individual-based household transmission model [58] to simulate the infection and fatality status among household member. For each household, we first simulate the number of household contacts (from 1-6).

Based on the above model, we can compute the number of secondary infections and also the number of fatality cases. Then we can use exact binomial method to compute the upper bound and lower bound of the estimates of CFR.

For each simulation, we have 1000 replications. We conducted simulations with CFR equal to 0.01%, 0.1%, 1%, 2%, 10% and 20% representing different levels of severity. We also conducted simulations with transmission probability equal to 5%, 10%, 15% and 20% for disease with different transmissibility in households.

We repeated the same simulation model to explore the impact of different severity in different groups (children vs adults as example) on sample size requirement. We assume the number of children and adults were equally

distributed, and the CFR for children was half for adults. We conduct the same set of value for transmission probability and CFR as described above.

Supplementary References

1. Arnedo-Pena A, Sabater-Vidal S, Meseguer-Ferrer N,et al. COVID-19 secondary attack rate and risk factors in household contacts in Castellon (Spain): preliminary report. Enfermedades Emergentes 2020;19(2):64-70. Accessed November 11, 2020. https://docisolation.prod.fire.glass/?guid=45f61a53-bdcc-40ab-ded8-dd9646aa077c.
2. Bi Q, Wu Y, Mei S, Ye C, Zou X, Zhang Z, et al. Epidemiology and transmission of COVID-19 in 391 cases and 1286 of their close contacts in Shenzhen, China: a retrospective cohort study. Lancet Infect Dis. 2020;20(8):911-9. Epub 2020/05/01. doi: 10.1016/s1473-3099(20)30287-5. PubMed PMID: 32353347; PubMed Central PMCID: PMCPMC7185944.
3. Bo Y, Fen G, Cao D, Cai Y, Qian L, Li W, et al. Epidemiological and clinical characteristics of 214 families with COVID-19 in Wuhan, China. Int J Infect Dis. 2021;105:113-9. Epub 2021/02/13. doi: 10.1016/j.ijid.2021.02.021. PubMed PMID: 33578019; PubMed Central PMCID: PMCPMC7872852.
4. Boddington NL, Charlett A, Elgohari S, Byers C, Coughlan L, Vilaplana TG, et al. Epidemiological and clinical characteristics of early COVID-19 cases, United Kingdom of Great Britain and Northern Ireland. Bull World Health Organ. 2021;99(3):178-89. Epub 2021/03/16. doi: 10.2471/BLT.20.265603. PubMed PMID: 33716340; PubMed Central PMCID: PMCPMC7941108.
5. Broccia MM, de Knegt V, Mills E, Moller A, Gnesin FF, Fischer T, et al. Household exposure to SARS-CoV-2 and association with COVID-19 severity: a Danish nationwide cohort study. Clin Infect Dis. 2021. Epub 2021/04/25. doi: 10.1093/cid/ciab340. PubMed PMID: 33893489.
6. Chaw L, Koh WC, Jamaludin SA, Naing L, Alikhan MF, Wong J. Analysis of SARS-CoV-2 Transmission in Different Settings, Brunei. Emerg Infect Dis. 2020;26(11):2598-606. Epub 2020/10/10. doi: 10.3201/eid2611.202263. PubMed PMID: 33035448; PubMed Central PMCID: PMCPMC7588541.
7. Chen P, Zhang Y, Wen Y, Guo J, Jia J, Ma Y, et al. Epidemiological and clinical characteristics of 136 cases of COVID-19 in main district of Chongqing. J Formos Med Assoc. 2020;119(7):1180-4. Epub 2020/05/11. doi: 10.1016/j.jfma.2020.04.019. PubMed PMID: 32386675; PubMed Central PMCID: PMCPMC7188645.

8. Chen Y, Yao H, Fu J, Shu Q, Chen Z, Wu N, et al. The low contagiousness and new A958D mutation of SARS-CoV-2 in children: An observational cohort study. Int J Infect Dis. 2021. Epub 2021/08/29. doi: 10.1016/j.ijid.2021.08.036. PubMed PMID: 34454120; PubMed Central PMCID: PMCPMC8384730.

9. Cheng HY, Jian SW, Liu DP, Ng TC, Huang WT, Lin HH, et al. Contact Tracing Assessment of COVID-19 Transmission Dynamics in Taiwan and Risk at Different Exposure Periods Before and After Symptom Onset. Jama Internal Medicine. 2020;180(9):1156-63. doi: 10.1001/jamainternmed.2020.2020. PubMed PMID: WOS:000571868600006.

10. Dawson P, Rabold EM, Laws RL, Conners EE, Gharpure R, Yin S, et al. Loss of Taste and Smell as Distinguishing Symptoms of Coronavirus Disease 2019. Clin Infect Dis. 2021;72(4):682-5. Epub 2020/06/21. doi: 10.1093/cid/ciaa799. PubMed PMID: 32562541; PubMed Central PMCID: PMCPMC7337666.

11. Dupraz J, Butty A, Duperrex O, Estoppey S, Faivre V, Thabard J, et al. Prevalence of SARS-CoV-2 in Household Members and Other Close Contacts of COVID-19 Cases: A Serologic Study in Canton of Vaud, Switzerland. Open Forum Infect Dis. 2021;8(7):ofab149. Epub 2021/07/27. doi: 10.1093/ofid/ofab149. PubMed PMID: 34307723; PubMed Central PMCID: PMCPMC8083624.

12. Freeman EE, McMahon DE, Lipoff JB, Rosenbach M, Kovarik C, Takeshita J, et al. Pernio-like skin lesions associated with COVID-19: A case series of 318 patients from 8 countries. J Am Acad Dermatol. 2020;83(2):486-92. Epub 2020/06/02. doi: 10.1016/j.jaad.2020.05.109. PubMed PMID: 32479979; PubMed Central PMCID: PMCPMC7260509.

13. Gomaa MR, El Rifay AS, Shehata M, Kandeil A, Nabil Kamel M, Marouf MA, et al. Incidence, household transmission, and neutralizing antibody seroprevalence of Coronavirus Disease 2019 in Egypt: Results of a community-based cohort. PLoS Pathog. 2021;17(3):e1009413. Epub 2021/03/12. doi: 10.1371/journal.ppat.1009413. PubMed PMID: 33705496; PubMed Central PMCID: PMCPMC7987187.

14. Hu P, Ma M, Jing Q, Ma Y, Gan L, Chen Y, et al. Retrospective study identifies infection related risk factors in close contacts during COVID-19 epidemic. Int J Infect Dis. 2021;103:395-401. Epub 2020/12/15. doi:

10.1016/j.ijid.2020.12.011. PubMed PMID: 33310026; PubMed Central PMCID: PMCPMC7832759.

15. Hu S, Wang W, Wang Y, Litvinova M, Luo K, Ren L, et al. Infectivity, susceptibility, and risk factors associated with SARS-CoV-2 transmission under intensive contact tracing in Hunan, China. Nat Commun. 2021;12(1):1533. Epub 2021/03/23. doi: 10.1038/s41467-021-21710-6. PubMed PMID: 33750783; PubMed Central PMCID: PMCPMC7943579.

16. Kuwelker K, Zhou F, Blomberg B, Lartey S, Brokstad KA, Trieu MC, et al. Attack rates amongst household members of outpatients with confirmed COVID-19 in Bergen, Norway: A case-ascertained study. Lancet Reg Health Eur. 2021;3:100014. Epub 2021/04/20. doi: 10.1016/j.lanepe.2020.100014. PubMed PMID: 33871470; PubMed Central PMCID: PMCPMC8009692 s laboratory. Mount Sinai has filed patent applications to protect that assay and has licensed its use to several companies. Mount Sinai is also commercializing the assay. All other authors declare no conflict of interest.

17. Li F, Li YY, Liu MJ, Fang LQ, Dean NE, Wong GWK, et al. Household transmission of SARS-CoV-2 and risk factors for susceptibility and infectivity in Wuhan: a retrospective observational study. Lancet Infect Dis. 2021. Epub 2021/01/22. doi: 10.1016/s1473-3099(20)30981-6. PubMed PMID: 33476567; PubMed Central PMCID: PMCPMC7833912.

18. Li W, Zhang B, Lu J, Liu S, Chang Z, Peng C, et al. Characteristics of Household Transmission of COVID-19. Clin Infect Dis. 2020;71(8):1943-6. Epub 2020/04/18. doi: 10.1093/cid/ciaa450. PubMed PMID: 32301964; PubMed Central PMCID: PMCPMC7184465.

19. Li J, Gong X, Wang Z, Chen R, Li T, Zeng D, et al. Clinical features of familial clustering in patients infected with 2019 novel coronavirus in Wuhan, China. Virus Res. 2020;286:198043. Epub 2020/06/06. doi: 10.1016/j.virusres.2020.198043. PubMed PMID: 32502551; PubMed Central PMCID: PMCPMC7265838.

20. Luo L, Liu D, Liao X, Wu X, Jing Q, Zheng J, et al. Contact Settings and Risk for Transmission in 3410 Close Contacts of Patients With COVID-19 in Guangzhou, China : A Prospective Cohort Study. Ann Intern Med. 2020;173(11):879-87. Epub 2020/08/14. doi: 10.7326/m20-2671. PubMed

PMID: 32790510; PubMed Central PMCID: PMCPMC7506769
www.acponline.org/authors/icmje/ConflictOfInterestForms.do?msNum=M20-2671.
21. Maltezou HC, Vorou R, Papadima K, Kossyvakis A, Spanakis N, Gioula G, et al. Transmission dynamics of SARS-CoV-2 within families with children in Greece: A study of 23 clusters. J Med Virol. 2021;93(3):1414-20. Epub 2020/08/09. doi: 10.1002/jmv.26394. PubMed PMID: 32767703; PubMed Central PMCID: PMCPMC7441283.
22. Martinez-Fierro ML, Ríos-Jasso J, Garza-Veloz I, Reyes-Veyna L, Cerda-Luna RM, Duque-Jara I, et al. The role of close contacts of COVID-19 patients in the SARS-CoV-2 transmission: an emphasis on the percentage of nonevaluated positivity in Mexico. Am J Infect Control. 2021;49(1):15-20. Epub 2020/10/10. doi: 10.1016/j.ajic.2020.10.002. PubMed PMID: 33035601; PubMed Central PMCID: PMCPMC7538376.
23. Miyahara R, Tsuchiya N, Yasuda I, Ko YK, Furuse Y, Sando E, et al. Familial Clusters of Coronavirus Disease in 10 Prefectures, Japan, February-May 2020. Emerg Infect Dis. 2021;27(3):915-8. Epub 2021/02/25. doi: 10.3201/eid2703.203882. PubMed PMID: 33622475; PubMed Central PMCID: PMCPMC7920650.
24. Reukers DFM, van Boven M, Meijer A, Rots N, Reusken C, Roof I, et al. High infection secondary attack rates of SARS-CoV-2 in Dutch households revealed by dense sampling. Clin Infect Dis. 2021. Epub 2021/04/07. doi: 10.1093/cid/ciab237. PubMed PMID: 33822007.
25. Salihefendic N, Zildzic M, Huseinagic H, Ahmetagic S, Salihefendic D, Masic I. Intrafamilial Spread of COVID-19 Infection Within Population in Bosnia and Herzegovina. Mater Sociomed. 2021;33(1):4-9. Epub 2021/05/21. doi: 10.5455/msm.2021.33.4-9. PubMed PMID: 34012342; PubMed Central PMCID: PMCPMC8116071.
26. Sami S, Turbyfill CR, Daniel-Wayman S, Shonkwiler S, Fisher KA, Kuhring M, et al. Community Transmission of SARS-CoV-2 Associated with a Local Bar Opening Event - Illinois, February 2021. MMWR Morb Mortal Wkly Rep. 2021;70(14):528-32. Epub 2021/04/09. doi: 10.15585/mmwr.mm7014e3. PubMed PMID: 33830981; PubMed Central PMCID: PMCPMC8030980 Journal

Editors form for disclosure of potential conflicts of interest. No potential conflicts of interest were disclosed.
27. Shi Q, Hu Y, Peng B, Tang X-J, Wang W, Su K, et al. Effective control of SARS-CoV-2 transmission in Wanzhou, China. Nature Medicine. 2020. doi: 10.1038/s41591-020-01178-5.
28. Soriano-Arandes A, Gatell A, Serrano P, Biosca M, Campillo F, Capdevila R, et al. Household SARS-CoV-2 transmission and children: a network prospective study. Clin Infect Dis. 2021. Epub 2021/03/13. doi: 10.1093/cid/ciab228. PubMed PMID: 33709135; PubMed Central PMCID: PMCPMC7989526.
29. Steinberg J, Kennedy ED, Basler C, Grant MP, Jacobs JR, Ortbahn D, et al. COVID-19 Outbreak Among Employees at a Meat Processing Facility - South Dakota, March-April 2020. MMWR Morb Mortal Wkly Rep. 2020;69(31):1015-9. Epub 2020/08/08. doi: 10.15585/mmwr.mm6931a2. PubMed PMID: 32759914; PubMed Central PMCID: PMCPMC7454899 Journal Editors form for disclosure of potential conflicts of interest. No potential conflicts of interest were disclosed.
30. Sun WW, Ling F, Pan JR, Cai J, Miao ZP, Liu SL, et al. [Epidemiological characteristics of COVID-19 family clustering in Zhejiang Province]. Zhonghua Yu Fang Yi Xue Za Zhi. 2020;54(6):625-9. Epub 2020/03/17. doi: 10.3760/cma.j.cn112150-20200227-00199. PubMed PMID: 32171192.
31. Thiel SL, Weber MC, Risch L, Wohlwend N, Lung T, Hillmann D, et al. Flattening the curve in 52 days: characterisation of the COVID-19 pandemic in the Principality of Liechtenstein - an observational study. Swiss Med Wkly. 2020;150:w20361. Epub 2020/10/27. doi: 10.4414/smw.2020.20361. PubMed PMID: 33105020.
32. Trunfio M, Longo BM, Alladio F, Venuti F, Cerutti F, Ghisetti V, et al. On the SARS-CoV-2 "Variolation Hypothesis": No Association Between Viral Load of Index Cases and COVID-19 Severity of Secondary Cases. Front Microbiol. 2021;12:646679. Epub 2021/04/06. doi: 10.3389/fmicb.2021.646679. PubMed PMID: 33815334; PubMed Central PMCID: PMCPMC8010676.
33. Ustundag G, Yilmaz-Ciftdogan D, Kara-Aksay A, Sahin A, Ekemen-Keles Y, Orsdemir-Hortu H, et al. COVID-19 in healthy children: What is the effect of household contact? Pediatr Int. 2021. Epub 2021/06/20. doi: 10.1111/ped.14890. PubMed PMID: 34145691.

34. Wang Z, Ma W, Zheng X, Wu G, Zhang R. Household transmission of SARS-CoV-2. J Infect. 2020;81(1):179-82. Epub 2020/04/14. doi: 10.1016/j.jinf.2020.03.040. PubMed PMID: 32283139; PubMed Central PMCID: PMCPMC7151261.

35. Wu J, Huang Y, Tu C, Bi C, Chen Z, Luo L, et al. Household Transmission of SARS-CoV-2, Zhuhai, China, 2020. Clin Infect Dis. 2020;71(16):2099-108. Epub 2020/05/12. doi: 10.1093/cid/ciaa557. PubMed PMID: 32392331; PubMed Central PMCID: PMCPMC7239243.

36. Wu P, Liu F, Chang Z, Lin Y, Ren M, Zheng C, et al. Assessing asymptomatic, pre-symptomatic and symptomatic transmission risk of SARS-CoV-2. Clin Infect Dis. 2021. Epub 2021/03/28. doi: 10.1093/cid/ciab271. PubMed PMID: 33772573.

37. Xie W, Chen Z, Wang Q, Song M, Cao Y, Wang L, et al. Infection and disease spectrum in individuals with household exposure to SARS-CoV-2: A family cluster cohort study. J Med Virol. 2021;93(5):3033-46. Epub 2021/02/05. doi: 10.1002/jmv.26847. PubMed PMID: 33538342; PubMed Central PMCID: PMCPMC8014049.

38. Zheng X, Luo S, Sun Y, Han M, Liu J, Sun L, Zhang L, Ling P, Ding Y, Jin T, Liu Z, Weng J. Asymptomatic patients and asymptomatic phases of coronavirus disease 2019 (COVID-19): a population-based surveillance study. . Natl Sci Rev. 2020;7(10), 1527–1539.

39. Chang TH, Wu JL, Chang LY. Clinical characteristics and diagnostic challenges of pediatric COVID-19: A systematic review and meta-analysis. J Formos Med Assoc. 2020;119(5):982-9. Epub 2020/04/21. doi: 10.1016/j.jfma.2020.04.007. PubMed PMID: 32307322; PubMed Central PMCID: PMCPMC7161491.

40. Chen X, Chen Z, Azman AS, Deng X, Sun R, Zhao Z, et al. Serological evidence of human infection with SARS-CoV-2: a systematic review and meta-analysis. Lancet Glob Health. 2021;9(5):e598-e609. Epub 2021/03/12. doi: 10.1016/s2214-109x(21)00026-7. PubMed PMID: 33705690; PubMed Central PMCID: PMCPMC8049592.

41. Ciapponi A, Bardach A, Comandé D, Berrueta M, Argento FJ, Rodriguez Cairoli F, et al. COVID-19 and pregnancy: An umbrella review of clinical

presentation, vertical transmission, and maternal and perinatal outcomes. PLoS One. 2021;16(6):e0253974. Epub 2021/06/30. doi: 10.1371/journal.pone.0253974. PubMed PMID: 34185807; PubMed Central PMCID: PMCPMC8241118.

42. Fathi M, Vakili K, Sayehmiri F, Mohamadkhani A, Hajiesmaeili M, Rezaei-Tavirani M, et al. The prognostic value of comorbidity for the severity of COVID-19: A systematic review and meta-analysis study. PLoS One. 2021;16(2):e0246190. Epub 2021/02/17. doi: 10.1371/journal.pone.0246190. PubMed PMID: 33592019; PubMed Central PMCID: PMCPMC7886178.

43. Islam MM, Poly TN, Walther BA, Yang HC, Wang CW, Hsieh WS, et al. Clinical Characteristics and Neonatal Outcomes of Pregnant Patients With COVID-19: A Systematic Review. Front Med (Lausanne). 2020;7:573468. Epub 2021/01/05. doi: 10.3389/fmed.2020.573468. PubMed PMID: 33392213; PubMed Central PMCID: PMCPMC7772992.

44. Li B, Zhang S, Zhang R, Chen X, Wang Y, Zhu C. Epidemiological and Clinical Characteristics of COVID-19 in Children: A Systematic Review and Meta-Analysis. Front Pediatr. 2020;8:591132. Epub 2020/11/24. doi: 10.3389/fped.2020.591132. PubMed PMID: 33224909; PubMed Central PMCID: PMCPMC7667131.

45. Panahi L, Amiri M, Pouy S. Clinical Characteristics of COVID-19 Infection in Newborns and Pediatrics: A Systematic Review. Arch Acad Emerg Med. 2020;8(1):e50. Epub 2020/05/23. PubMed PMID: 32440661; PubMed Central PMCID: PMCPMC7212072.

46. Wong CKH, Wong JYH, Tang EHM, Au CH, Wai AKC. Clinical presentations, laboratory and radiological findings, and treatments for 11,028 COVID-19 patients: a systematic review and meta-analysis. Sci Rep. 2020;10(1):19765. Epub 2020/11/15. doi: 10.1038/s41598-020-74988-9. PubMed PMID: 33188232; PubMed Central PMCID: PMCPMC7666204.

47. Cui X, Zhao Z, Zhang T, Guo W, Guo W, Zheng J, et al. A systematic review and meta-analysis of children with coronavirus disease 2019 (COVID-19). J Med Virol. 2021;93(2):1057-69. Epub 2020/08/08. doi: 10.1002/jmv.26398. PubMed PMID: 32761898; PubMed Central PMCID: PMCPMC7436402.

48. Hashan MR, Smoll N, King C, Ockenden-Muldoon H, Walker J, Wattiaux A, et al. Epidemiology and clinical features of COVID-19 outbreaks in aged care facilities: A systematic review and meta-analysis. EClinicalMedicine. 2021;33:100771. Epub 2021/03/09. doi: 10.1016/j.eclinm.2021.100771. PubMed PMID: 33681730; PubMed Central PMCID: PMCPMC7917447.
49. Wang K, Wu C, Xu J, Zhang B, Zhang X, Gao Z, et al. Factors affecting the mortality of patients with COVID-19 undergoing surgery and the safety of medical staff: A systematic review and meta-analysis. EClinicalMedicine. 2020;29:100612. Epub 2020/11/11. doi: 10.1016/j.eclinm.2020.100612. PubMed PMID: 33169112; PubMed Central PMCID: PMCPMC7641595.
50. Ip DK, Lau LL, Chan KH, Fang VJ, Leung GM, Peiris MJ, et al. The Dynamic Relationship Between Clinical Symptomatology and Viral Shedding in Naturally Acquired Seasonal and Pandemic Influenza Virus Infections. Clin Infect Dis. 2015. doi: 10.1093/cid/civ909. PubMed PMID: 26518469.
51. Papenburg J, Baz M, Hamelin ME, Rheaume C, Carbonneau J, Ouakki M, et al. Household transmission of the 2009 pandemic A/H1N1 influenza virus: elevated laboratory-confirmed secondary attack rates and evidence of asymptomatic infections. Clin Infect Dis. 2010;51(9):1033-41. doi: 10.1086/656582. PubMed PMID: 20887206.
52. Suess T, Buchholz U, Dupke S, Grunow R, an der Heiden M, Heider A, et al. Shedding and transmission of novel influenza virus A/H1N1 infection in households--Germany, 2009. Am J Epidemiol. 2010;171(11):1157-64. doi: 10.1093/aje/kwq071. PubMed PMID: 20439308.
53. Cauchemez S, Fraser C, Van Kerkhove MD, Donnelly CA, Riley S, Rambaut A, et al. Middle East respiratory syndrome coronavirus: quantification of the extent of the epidemic, surveillance biases, and transmissibility. Lancet Infect Dis. 2014;14(1):50-6. Epub 2013/11/19. doi: 10.1016/S1473-3099(13)70304-9. PubMed PMID: 24239323; PubMed Central PMCID: PMCPMC3895322.
54. Assiri A, Abedi GR, Bin Saeed AA, Abdalla MA, al-Masry M, Choudhry AJ, et al. Multifacility Outbreak of Middle East Respiratory Syndrome in Taif, Saudi Arabia. Emerg Infect Dis. 2016;22(1):32-40. Epub 2015/12/23. doi: 10.3201/eid2201.151370. PubMed PMID: 26692003; PubMed Central PMCID: PMCPMC4696715.

55. Alsahafi AJ, Cheng AC. The epidemiology of Middle East respiratory syndrome coronavirus in the Kingdom of Saudi Arabia, 2012-2015. Int J Infect Dis. 2016;45:1-4. Epub 2016/02/16. doi: 10.1016/j.ijid.2016.02.004. PubMed PMID: 26875601; PubMed Central PMCID: PMCPMC7110824.
56. Penttinen PM, Kaasik-Aaslav K, Friaux A, Donachie A, Sudre B, Amato-Gauci AJ, et al. Taking stock of the first 133 MERS coronavirus cases globally--Is the epidemic changing? Euro Surveill. 2013;18(39). Epub 2013/10/08. doi: 10.2807/1560-7917.es2013.18.39.20596. PubMed PMID: 24094061.
57. Sha J, Li Y, Chen X, Hu Y, Ren Y, Geng X, et al. Fatality risks for nosocomial outbreaks of Middle East respiratory syndrome coronavirus in the Middle East and South Korea. Arch Virol. 2017;162(1):33-44. Epub 2016/09/25. doi: 10.1007/s00705-016-3062-x. PubMed PMID: 27664026; PubMed Central PMCID: PMCPMC7087023.
58. Jing QL, Liu MJ, Zhang ZB, Fang LQ, Yuan J, Zhang AR, et al. Household secondary attack rate of COVID-19 and associated determinants in Guangzhou, China: a retrospective cohort study. Lancet Infect Dis. 2020;20(10):1141-50. Epub 2020/06/21. doi: 10.1016/S1473-3099(20)30471-0. PubMed PMID: 32562601; PubMed Central PMCID: PMCPMC7529929.
59. He X, Lau EHY, Wu P, Deng X, Wang J, Hao X, et al. Temporal dynamics in viral shedding and transmissibility of COVID-19. Nature Medicine. 2020;26(5):672-5. doi: 10.1038/s41591-020-0869-5.
60. Li Q, Guan X, Wu P, Wang X, Zhou L, Tong Y, et al. Early Transmission Dynamics in Wuhan, China, of Novel Coronavirus-Infected Pneumonia. N Engl J Med. 2020. doi: 10.1056/NEJMoa2001316. PubMed PMID: 31995857.
